# Supplementary material for: Mertk Interacts with Tim-4 to Enhance Tim-4-Mediated Efferocytosis
Source: Cells. 2020 Jul 6;9(7):1625. doi: 10.3390/cells9071625 (PMC7408610; doi:10.3390/cells9071625)
Supplement: Supplementary file 1 [file cells-09-01625-s001.pdf]

Supplementary Figures

Mertk interacts with Tim-4 to enhance Tim-4-mediated efferocytosis

Byeongjin Moon[1, 2], Juyeon Lee[1, 2], Sang-Ah Lee[1, 2], Chanhyuk Min[1, 2], Hyunji Moon[1, 2], Deokhwan Kim[1, 2], Susumin Yang[1, 2], Heera Moon[1], Jaeseon Jeon[1], Young-Eun Joo[3], Daeho Park[1, 2, *]

[1] School of Life Sciences, Gwangju Institute of Science and Technology, Gwangju 61005, Korea; byeongjinmoon@gist.ac.kr (B.M.); iris260@gist.ac.kr (J.L.); sanga03@gist.ac.kr (S-A.L.); alscksgur@gist.ac.kr (C.M.); hjmoon311@gist.ac.kr (H.M.); po7322@gist.ac.kr (D.K.); susuminy@gist.ac.kr (S.Y.); gmlfk301@gist.ac.kr (H.M.); jeonjaeseon17@gist.ac.kr (J.J.); yejoo@chonnam.ac.kr (Y-E.J.)

[2] Center for Cell Mechanobiology, Gwangju Institute of Science and Technology, Gwangju 61005, Korea

[3] Department of Internal Medicine, Chonnam National Univerity, Gwangju 61469, Korea

* Correspondence: daehopark@gist.ac.kr (D.P.) ; Tel.: 82-62-715-2890;

*** Correspondence to**

Daeho Park

School of Life Sciences

Gwangju Institute of Science and Technology

Gwangju 61005, Korea

Tel.: 82-62-715-2890

Fax: 82-62-715-2484

E-mail: daehopark@gist.ac.kr

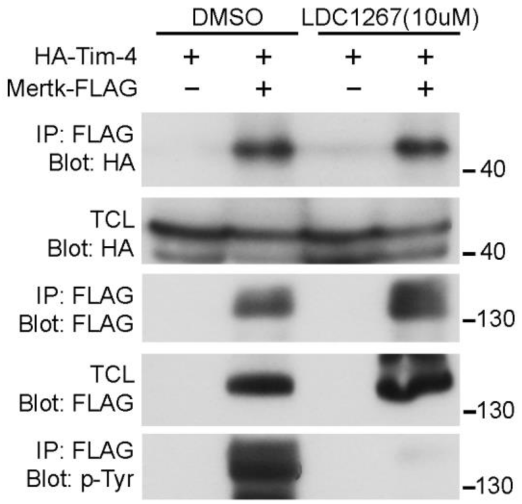

Supplementary Figure 1

293T cells were transfected with the indicated plasmids. 2 d after transfection, the cells were treated with the indicated concentration of LDC1267 for 24 h. Then the cells were lysed, and FLAG-tagged Mertk was precipitated with anti-FLAG antibody-conjugated agarose beads. Bound proteins were detected with the indicated antibodies

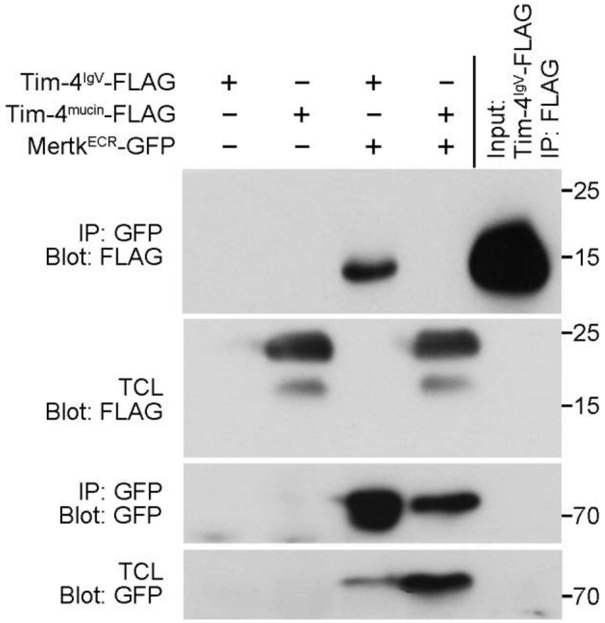

Supplementary Figure 2

The lysates of 293T cells transfected with the indicated plasmids were incubated with an anti-GFP antibody and protein A/G agarose beads (lane 1-4) or anti-FLAG antibody-conjugated agarose beads (lane 5). Bound proteins were detected by immunoblotting. Input: the immunoprecipitants of the lysates of 293T cells transfected with Tim-4^{IgV}-FLAG by an anti-FLAG antibody.

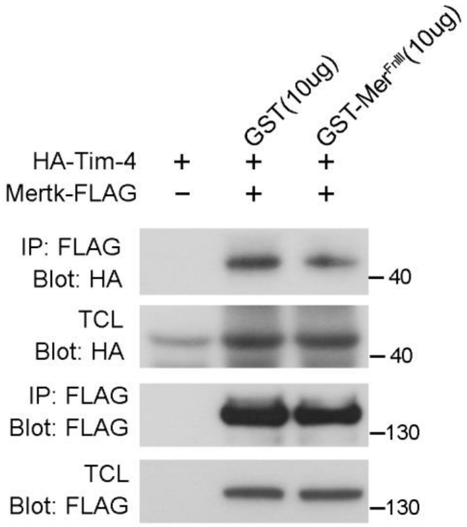

Supplementary Figure 3

The lysates of 293T cells transfected with the indicated plasmids were incubated with anti-FLAG antibody-conjugated agarose beads in the presence of purified GST-Mertk^{FnIII} or GST. Bound proteins on the beads were detected by immunoblotting.

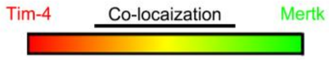

$$\text{Co-localization index} = \frac{\text{No. of pixel with the co-localization color}}{\text{No. of total pixels}}$$

	GST				
Image	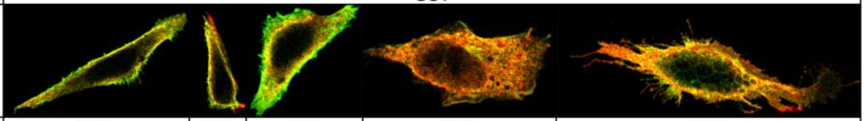				
Co-localization index	0.74	0.71	0.62	0.47	0.63
	GST-Mertk^{FnIII}				
Image	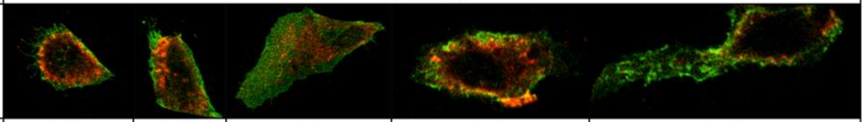				
Co-localization index	0.35	0.34	0.38	0.42	0.33

Supplementary Figure 4

The formula for the co-localization index is the number of pixels with a co-localization color divided by the number of total pixels (the formula shown above). The range of two colors from green (Mertk) to red (Tim-4) was set (shown top), and the pixels in the middle range of the color (yellowish) was counted using ImageJ. Representative images and their co-localization indexes are shown.

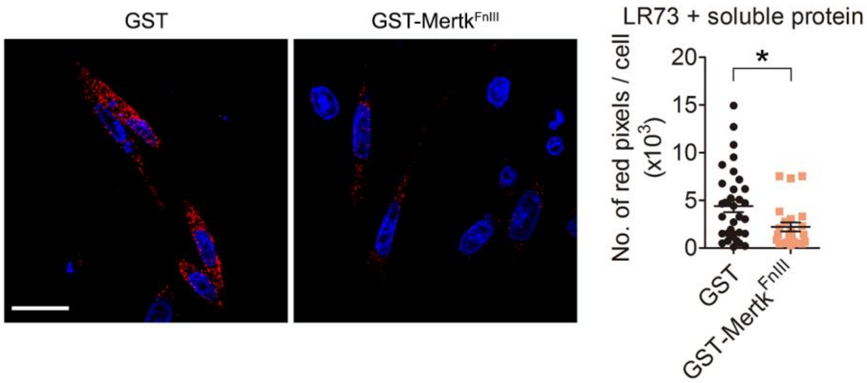

Supplementary Figure 5

A proximity ligation assay was performed using LR73 cells transfected with the indicated plasmids. 1 d after transfection, the cells were incubated with GST or GST-Mertk^{FnIII} for 2 h, fixed, blocked, and incubated with anti-FLAG and anti-Tim-4^{tail} antibodies. The cells then were incubated with the amplification solution at 37 °C overnight. Images were acquired by confocal microscopy and quantified (F, n≥24 cells). Scale bar, 40 μm.
